# The Hellenic Emergency Laparotomy Study (HELAS): A Prospective Multicentre Study on the Outcomes of Emergency Laparotomy in Greece

**DOI:** 10.1007/s00268-022-06723-6

**Published:** 2022-09-15

**Authors:** Konstantinos Lasithiotakis, Evangelos I. Kritsotakis, Stamatios Kokkinakis, Georgia Petra, Konstantinos Paterakis, Garyfallia-Apostolia Karali, Vironas Malikides, Charalampos S. Anastasiadis, Odysseas Zoras, Nikolas Drakos, Ioannis Kehagias, Dimitrios Kehagias, Nikolaos Gouvas, Georgios Kokkinos, Ioanna Pozotou, Panayiotis Papatheodorou, Kyriakos Frantzeskou, Dimitrios Schizas, Athanasios Syllaios, Ifaistion M. Palios, Konstantinos Nastos, Markos Perdikaris, Nikolaos V. Michalopoulos, Ioannis Margaris, Evangelos Lolis, Georgia Dimopoulou, Dimitrios Panagiotou, Vasiliki Nikolaou, Georgios K. Glantzounis, George Pappas-Gogos, Kostas Tepelenis, Georgios Zacharioudakis, Savvas Tsaramanidis, Ioannis Patsarikas, Georgios Stylianidis, Georgios Giannos, Michael Karanikas, Konstantinia Kofina, Markos Markou, Emmanuel Chrysos

**Affiliations:** 1 Department of General Surgery, School of Medicine, University Hospital of Heraklion, University of Crete, Heraklion, Crete, Greece; 2 Laboratory of Biostatistics, School of Medicine, University of Crete, Heraklion, Crete, Greece; 3 Department of Surgical Oncology, School of Medicine, University Hospital of Heraklion, University of Crete, Heraklion, Crete, Greece; 4 Department of Surgery, School of Medicine, University General Hospital of Patras, University of Patras, Patras, Greece; 5 Department of Surgery, School of Medicine, General Hospital of Nicosia, University of Cyprus, Nicosia, Cyprus; 6 First Department of Surgery, Laikon General Hospital, National and Kapodistrian University of Athens, Athens, Greece; 7 Second Propaedeutic Department of Surgery, Laikon General Hospital, National and Kapodistrian University of Athens, Athens, Greece; 8 Department of Surgery, School of Medicine, University General Hospital Attikon, University of Athens, Athens, Greece; 9 Department of Surgery, General Hospital of Volos, Volos, Greece; 10 Department of Surgery, General Hospital of Trikala, Trikala, Greece; 11 Department of Surgery, University Hospital of Ioannina, Ioannina, Greece; 12 Department of Surgery, School of Medicine, Ippokrateio General Hospital of Thessaloniki, Aristotle University of Thessaloniki, Thessaloniki, Greece; 13 2nd Department of Surgery, Evangelismos General Hospital, Athens, Greece; 14 Department of Surgery, School of Medicine, University General Hospital of Alexandroupolis, University of Thrace, Alexandroupolis, Greece; 15 Department of General Surgery, University Hospital of Crete, 71110 Heraklion, Greece

## Abstract

**Background:**

Emergency laparotomy (EL) is accompanied by high post-operative morbidity and mortality which varies significantly between countries and populations. The aim of this study is to report outcomes of emergency laparotomy in Greece and to compare them with the results of the National Emergency Laparotomy Audit (NELA).

**Methods:**

This is a multicentre prospective cohort study undertaken between 01.2019 and 05.2020 including consecutive patients subjected to EL in 11 Greek hospitals. EL was defined according to NELA criteria. Demographics, clinical variables, and post-operative outcomes were prospectively registered in an online database. Multivariable logistic regression analysis was used to identify independent predictors of post-operative mortality.

**Results:**

There were 633 patients, 53.9% males, ASA class III/IV 43.6%, older than 65 years 58.6%. The most common operations were small bowel resection (20.5%), peptic ulcer repair (12.0%), adhesiolysis (11.8%) and Hartmann’s procedure (11.5%). 30-day post-operative mortality reached 16.3% and serious complications occurred in 10.9%. Factors associated with post-operative mortality were increasing age and ASA class, dependent functional status, ascites, severe sepsis, septic shock, and diabetes. HELAS cohort showed similarities with NELA patients in terms of demographics and preoperative risk. Post-operative utilisation of ICU was significantly lower in the Greek cohort (25.8% vs 56.8%) whereas 30-day post-operative mortality was significantly higher (16.3% vs 8.7%).

**Conclusion:**

In this study, Greek patients experienced markedly worse mortality after emergency laparotomy compared with their British counterparts. This can be at least partly explained by underutilisation of critical care by surgical patients who are at high risk for death.

**Supplementary Information:**

The online version contains supplementary material available at 10.1007/s00268-022-06723-6.

## Introduction

Emergency laparotomy (EL) is one of the most common procedures performed in emergency surgery. It is associated with substantial mortality ranging between 10 and 55% in studies using variable inclusion criteria in their samples [1–4]. Under the term emergency laparotomy, hundreds of surgical procedures can be included in the treatment of an even larger number of diseases reflecting a highly diverse patient cohort [5, 6]. This fact, along with the limited time for preoperative optimisation, the poor functional and physiological reserves of the patients and the unpreparedness of the health systems to manage this heterogeneous population are the most common reasons for the poor outcomes. The unsatisfactory standards of care of emergency surgical admissions compared with elective operations have drawn the attention of the surgical professional bodies in western countries after the millennium [4, 5]. During the last decade, there is mounting evidence that data-driven quality improvement interventions can lead to higher adherence to defined standards and perhaps to improved outcomes such as death rates at a national level [7–9]. However, most of the evidence comes from studies derived from the UK and the USA [4, 7, 10, 11]. The generalisation of the findings of these studies to other countries is limited due to the significant variation of the outcomes and relevant standards of care between hospitals and countries [12, 13]. Therefore, the aim of this study is to present prospectively collected morbidity and mortality outcomes of emergency laparotomy in a multicentre setting and to compare them with international benchmark rates from the literature.

## Patients and methods

The study was approved by the Institutional Review Board and the Bioethics Committee of the University Hospital of Crete (No. 1681). The manuscript has been written following the STROBE recommendations for reporting observational studies (Supplementary Table S1) [14]. This is a prospective multicentre cohort study with consecutive data collection. 10 hospitals from Greece and 1 from Cyprus contributed to the cohort. There were 1 secondary, 2 tertiary hospitals and 8 university hospitals providing emergency general surgery care to the general local population. Participating hospitals were invited to submit prospective anonymised data on patients undergoing emergency laparotomy. All patients who had an emergency laparotomy between 01.2020 and 05.2021 were eligible for inclusion. Detailed inclusion and exclusion criteria are shown in Supplement file and agree with the National Emergency Laparotomy Audit (NELA) study to enable a meaningful comparison of our cohort with data published in the 7th NELA report (year 2020–2021) [15]. Briefly, appendectomies, negative diagnostic laparoscopies, biopsy procedures, and non-gastrointestinal (GI) surgery, elective GI surgery and those aged less than 18 years were not included. If a patient had more than one emergency laparotomy, only data from the first procedure were analysed. Registered data included patients’ demographics, clinical variables, anatomical site of surgery, operative procedure, duration post-operative care in the Intensive Care Unit (ICU), post-operative length of stay and the occurrence of any post-operative complication as defined and graded by Dindo et al. [16]. The definitions of variables and post-operative outcomes are presented in the Supplement Table S2. The study size was determined by the number of eligible patients in the participating hospital over the study period and no a priori calculation of sample size was performed.

### Statistical analysis

Categorical data were presented as frequencies and proportions (%). Numerical data were summarised as mean with standard deviation or median with interquartile range (IQR) depending on the degree of skewness in the distributions. Confidence intervals (CI) were estimated based on the binomial distribution with the Clopper–Pearson method for proportions and the Mood–Graybill method for medians.

Mixed-effects logistic regression analysis was used to assess independent risk factors of 30-day post-operative death, with hospital entered as random intercept. A set of 19 preoperative variables were selected based on clinical judgement and literature review. The direction and strength of associations were summarised using odds ratios (OR) with respective Wald CΙ based on the Normal distribution. Multicollinearity was ruled out by examining variance inflation factors. Nonlinearity in the log (odds) for continuous variables (age and Body Mass Index (BMI)) was assessed using locally weighted smoothed (lowess) scatterplots and modelled with restricted cubic splines. Missing values ranged between 0 and 1.3% for individual variables, and a complete case analysis would remove 4.4% of the data. Multiple imputation (10 iterations) by chained equations was used to handle the missing data. An initial multivariable logistic regression model was constructed containing all variables with two-sided *p* < 0.25 on univariable regression. Variables that did not contribute at the traditional level of significance (*p* < 0.05) were eliminated from the initial multivariable model unless there was evidence of confounding (change in any model coefficient by at least 20%) [17]. Variables that did not enter the initial model (*p* > 0.25 on univariable analysis) were forced sequentially into the model to examine the possibility of negative confounding. Predetermined criteria to retain a variable into the final main-effects model were two-sided *p* < 0.05 and/or substantial confounding effect. Data were processed and analysed using STATA v.17 (StataCorp, College Station, TX, USA). Stata’s commands mi and meqrlogit were used for multiple imputation and mixed-effects logistic regression, respectively.

## Results

A total of 633 patients were included. Rates of missing data were less than 2% for all variables except for the variable “timeliness of surgery” where the missing data rate was 6.4%. Table 1 displays the clinical characteristics of the patients stratified by 30-day post-operative mortality. The most common indications for emergency laparotomy were gastrointestinal obstruction 247 (39.0%), perforation 226 (35.7%) and ischaemia 94 (14.8%). 308 (48.1%) patients had a COVID test at the time of their admission and 9 (1.4%) were positive. The post-operative outcomes are displayed in Table 2. An uneventful recovery was observed in 282 (44.5%). Serious complications (III–IV) were observed in 69 (10.9%) of the patients and 55 (8.7%) of them returned to the operating theatre because of a complication. The rate of post-operative death within 30-days was 16.3%. Mortality associated with preoperative diagnosis and post-operative complications are displayed in Supplementary Table S3 and S4, respectively. After the operation 511 (80.7%) of the patients were directly transferred to the ward, 100 (15.8%) were admitted in the ICU and 22 (3.5%) required ICU treatment after initial hospitalisation in the ward (Table 2). Among non-survivors, 45 (43.7%) died in the ward without ICU treatment (Supplementary Table S5). In the present study, there were 22 patients who had an unplanned admission in the ICU, 18 of them (81%) were at high or very high post-operative risk and 13 (59.1%) died post-operatively (Supplementary Table S6). Finally, there were 45 patients who died post-operatively and received no ICU treatment during their hospital stay. 84.7% of them were at high or very high risk according to NELA risk calculator (Supplementary Table S5-S7).

**Table 1:** Demographics and clinical characteristics according to 30-day post-operative mortality in patients undergoing emergency laparotomy in 11 Greek Hospitals, 2019–2020

Risk factor	No. (%) of patients or mean ± SD			Crude (unadjusted) effect		
	Full cohort (*n* = 633)	Survived (*n* = 530)	Died (*n* = 103)	OR	95% CI	*p*
Age, years	66.2 ± 16.7	64.3 ± 16.8	75.8 ± 12.4	1.75^1^	1.47–2.09	<0.001
Male sex	341 (53.9)	287 (54.4)	53 (51.5)	0.92	0.60–1.41	0.698
BMI, kgs/m^2^	26.5 ± 5.3	26.5 ± 5.1	26.6 ± 6.3			
BMI (kgs/m^2^), RCS term 1^2^				0.80	0.68–0.95	0.044
BMI (kgs/m^2^), RCS term 2^2^				2.19	1.00–4.78	
BMI (kgs/m^2^), RCS term 2^2^				0.11	0.01–1.42	
ASA class						<0.001
I	150 (23.7)	145 (27.5)	5 (4.9)	1.00		
II	201 (31.8)	188 (35.7)	12 (11.7)	0.24	0.13–0.44	
III	171 (27.1)	141 (26.8)	30 (29.1)	1.12	0.70–1.80	
IV	104 (16.5)	52 (9.9)	51 (49.5)	9.10	5.63–14.72	
V	6 (0.9)	1 (0.2)	5 (4.9)	26.89	3.09–234.33	
Preoperative functional status						<0.001
Independent	444 (70.4)	402 (76.3)	41 (40.2)	1.00		
Partially dependent	154 (24.4)	105 (19.9)	49 (48.0)	4.07	2.56–6.47	
Totally dependent	33 (5.2)	20 (3.8)	12 (11.8)	3.66	1.72–7.79	
Chronic steroid use	55 (8.8)	38 (7.3)	16 (15.7)	2.47	1.32–4.62	0.005
Ascites^3^	81 (12.8)	54 (10.2)	27 (26.2)	3.09	1.84–5.21	<0.001
Anticipated severity of malignancy						0.141
None	462 (73.1)	394 (74.8)	67 (65.0)	1.00		
Primary	78 (12.3)	63 (12.0)	15 (14.6)	1.28	0.69–2.36	
Nodal metastasis	19 (3.0)	14 (2.7)	5 (4.9)	1.87	0.65–5.37	
Distant metastasis	73 (11.6)	56 (10.6)	16 (15.5)	1.64	0.90–2.98	
Diabetes mellitus	103 (16.3)	72 (13.6)	31 (30.4)	2.76	1.68–4.51	<0.001
Cardiac comorbidity	264 (41.8)	204 (38.7)	60 (58.8)	2.32	1.48–3.62	<0.001
Borderline cardiomegaly chest X-ray	38 (6.0)	33 (6.3)	5 (4.9)	0.77	0.29–2.05	0.606
	30 (4.7)	22 (4.2)	8 (7.8)	2.02	0.85–4.81	0.111
Respiratory History						0.003
No dyspnoea	570 (91.2)	485 (92.9)	84 (83.2)	1.00		
Dyspnoea on exertion or limiting	36 (5.8)	24 (4.6)	12 (11.9)	3.09	1.44–6.64	
Dyspnoea at rest or long-term oxygen therapy	19 (3.0)	13 (2.5)	5 (5.0)	2.56	0.88–7.46	
Smoking^4^	186 (29.4)	165 (31.3)	20 (19.4)	0.52	0.31–0.88	0.015
Haemodialysis or CVVH	10 (1.6)	6 (1.1)	4 (3.9)	3.29	0.91–11.93	0.070
Preoperative acute renal failure	77 (12.2)	50 (9.5)	27 (26.5)	3.66	2.10–6.36	<0.001
Sepsis^5^						<0.001
None	309 (48.8)	284 (53.8)	24 (23.3)	1.00		
Two SIRS criteria	218 (34.4)	186 (35.2)	32 (31.1)	0.84	0.53–1.33	
Severe Sepsis	81 (12.8)	48 (9.1)	32 (31.1)	4.64	2.76–7.80	
Septic Shock	25 (3.9)	10 (1.9)	15 (14.6)	9.40	3.99–22.15	
Preoperative diagnosis						0.040
Perforation	226 (35.7)	187 (35.4)	38 (36.9)	1.00		
Obstruction	247 (39.0)	218 (41.3)	29 (28.2)	0.55	0.34–0.87	
Ischaemia	94 (14.8)	74 (14.0)	20 (19.4)	1.45	0.84–2.52	
Other	66 (10.4)	49 (9.3)	16 (15.5)	1.75	0.95–3.22	
Operation type						0.163
Adhesiolysis	75 (11.8)	72 (13.6)	3 (2.9)	1.00		
Small bowel resection	130 (20.5)	106 (20.1)	24 (23.3)	1.21	0.73–2.01	
Colectomy right	58 (9.2)	48 (9.1)	10 (9.7)	1.09	0.53–2.24	
Hartmann's procedure	73 (11.5)	59 (11.2)	14 (13.6)	1.21	0.64–2.29	
Strangulated hernia with bowel resection	38 (6.0)	35 (6.6)	3 (2.9)	0.42	0.13–1.41	
Peptic ulcer repair	76 (12.0)	63 (11.9)	12 (11.7)	1.06	0.55–2.04	
Colectomy other	50 (7.9)	38 (7.2)	12 (11.7)	1.70	0.85–3.38	
Stoma formation	41 (6.5)	33 (6.3)	8 (7.8)	1.25	0.56–2.81	
Other	92 (14.5)	74 (14.0)	17 (16.5)	1.18	0.66–2.10	

SD, standard deviation; OR, odds ratio, CI confidence interval; ASA, American Society of Anaesthesiologists; SIRS, systemic inflammatory response syndrome; CVP, central venous pressure; CVVH, continuous veno-venous haemofiltration; BMI, body mass index; RCS, restricted cubic spline

^1^Effect per 10 years increase in age

^2^BMI was modelled using restricted cubic splines with slopes defined at the 5th, 35th, 65th and 95th

^3^Ascites within 30 days before surgery

^4^Smoking during the 12 months prior to surgery

^5^Sepsis within 48 h from surgery percentiles. The numeric results for the BMI splines are not directly interpretable. The resulting model fit is graphically shown in Supplementary Figure S1

**Table 2:** Post-operative outcomes of patients undergoing emergency laparotomy in 11 surgical centres in Greece, 2019–2020

Post-operative outcome	No. of patients	Proportion (%) or median	95% confidence interval
30-day death	103	16.3%	13.5–19.4%
Return to theatre	55	8.7%	6.6–11.2%
Complication severity grade			
I	89	14.1%	11.5–17.0%
II	90	14.2%	11.6–17.2%
III	42	6.6%	4.8–8.9%
IV	27	4.3%	2.8–6.2%
Sepsis	90	14.3%	11.6–17.2%
Septic shock	63	10.0%	7.7–12.6%
Bleeding requiring transfusion	38	6.0%	4.3–8.2%
Cardiac arrest	56	8.9%	6.8–11.4%
Pneumonia	45	7.1%	5.2–9.4%
Pulmonary embolism	4	0.6%	0.2–1.6%
Stroke	1	0.2%	0.0–0.9%
Acute kidney injury	45	7.1%	5.2–9.4%
Myocardial infarction	4	0.6%	0.2–1.6%
Surgical site infection			
Superficial incisional	46	59.0%	47.3–70.0%
Deep incisional	20	25.6%	16.4–36.8%
Organ/space	12	15.4%	8.2–25.3%
Deep venous thrombosis	1	0.2%	0.0–0.9%
Urinary tract infection	5	0.8%	0.3–1.8%
Delirium	19	3.0%	1.8–4.7%
Other complication	83	13.2%	10.6–16.1%
Functional status at discharge			
Independent	369	69.6%	65.5–73.5%
Partially dependent	110	20.8%	17.4–24.5%
Totally dependent	51	9.6%	7.2–12.5%
ICU length of stay (days)	119	7	5.0–9.7
Hospital length of stay (days)	606	10	9.0–10.0

Complication severity is according to Clavien-Dindo classification and refers to the most severe complication in case of multiple complications, ICU, intensive care unit

**Fig. 1:**
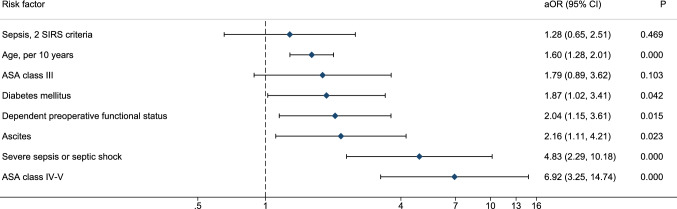
Independent risk factors of 30-day post-operative mortality. The forest plot depicts adjusted odds ratios (aOR) as diamonds and 95% confidence intervals (CI) as horizontal lines on a logarithmic scale. The reference category for the American Society of Anaesthesiologists (ASA) class is the I–II class. The reference category for the sepsis grades is absence of sepsis. Reported effects were estimated by multivariable mixed-effects logistic regression with hospital entered as random intercept. The final set of risk factors was selected following purposeful variable selection from 19 candidate preoperative factors

In the univariate analysis, factors significantly associated with post-operative death are presented in Table 1. In the multivariable analysis, the independent preoperative predictors of mortality were increasing age, ASA class 4–5, dependent preoperative functional status, ascites, severe sepsis or septic shock and diabetes (Fig. 1 and Supplementary Table S8). The comparative analysis of our data with those of the 7th NELA report is depicted in Table 3. The distribution of age and gender did not differ significantly between studies. Cross-tabulation of the two cohorts according to the preoperative NELA risk showed comparable risk between the two cohorts (Table 3). Post-operatively markedly higher rates of NELA patients were admitted directly in the ICU (56.8% vs. 15.8%). Among 91 patients who were transferred to the ICU immediately after surgery, 76% were at high or very high risk for death (> 5%) and 40 (51.3%) died within 30-days (Supplementary Table S5 and S7). Post-operative 30-day mortality was markedly higher in this study compared with the contemporary 7th NELA report (16.3% vs. 8.7%). Finally, case ascertainment rate exceeded 90% in 7 out of 11 centres (63.6%) and ranged between 80 and 90% in 4 out of 11 centres (36.4%) (Table 4).

**Table 3:** Comparison of the data of the Hellenic Emergency Laparotomy Study with the 7th report of the National Emergency Laparotomy Audit (NELA) from the United Kingdom

	HELAS *N* (%)	7th NELA *N* (%)
Gender		
Male	341 (53.9%)	10,740 (49.2)
Female	292 (46.1%)	11,106 (50.8)
Age groups		
< 55	154 (24.3%)	5862 (26.8%)
56–65	108 (17.1%)	3886 (17.8%)
> 65	371 (58.6%)	12,098 (55.4%)
Sepsis within 48 h preop		
No	309 (48.8%)	14,125 (58.6%)
Yes	324 (52.2%)	6965 (42.4%)
Presence of malignancy	170 (26.8%)	4292 (19.6%)
ASA class		
I	150 (23.7%)	1923 (8.8)
II	201 (31.8%)	8139 (37.3)
III	171 (27.0%)	8294 (38.0)
IV	104 (16.4%)	3236 (14.8)
V	6 (0.9%)	254 (1.2)
Type of admission		
Urgent	602 (95.7%)	20,840 (95.4%)
Elective	27 (4.3%)	1006 (4.6%)
Urgency of operation		
Expedited (> 18 h)	71 (11.2%)	3678 (16.8)
Urgent (6–18 h)	179 (28.3%)	7537 (34.5)
Urgent (2–6 h)	206 (32.5%)	8409 (38.5)
Immediate (< 2 h)	177 (28.0%)	2218 (10.2)
Timeliness of arrival in theatre		
Urgent (6–18 h)	176 (92.6%)	5063 (79.7%)
Urgent (2–6 h)	142 (84.5%)	6386 (85.2%)
Immediate (< 2 h)	50 (82%)	1369 (68.4%)
CT preoperatively	538 (85.1%)	20,202 (92.5%)
Preoperative risk assessment		
< 5%	333 (52.6%)	NA (54.7%)
5–9.9%	102 (16.1%)	NA (17.0%)
10–24.9%	105 (16.6%)	NA (17.4%)
25–49.9%	63 (10.0%)	NA (7.9%)
≥ 50%	22 (3.5%)	NA (2.2%)
Missing	8 (1.3%)	NA (0.8%)
Procedure		
Adhesiolysis	75 (11.8%)	4109 (18.8%)
Small bowel resection	130 (20.5%)	3163 (14.5%)
Colectomy right	58 (9.2%)	3000 (13.7%)
Colectomy other	50 (7.9%)	2023 (9.3%)
Hartman’s procedure	73 (11.5%)	2800 (12.8%)
Peptic ulcer perf repair	76 (12.0%)	1043 (4.8%)
Stoma formation	41 (6.5%)	954 (4.4%)
Other	92 (14.5%)	4754 (21.8%)
Post-operative pathway		
Ward care	511 (80.7%)	7890 (36.1%)
Ward care prior ICU	22 (3.5%)	698 (3.2%)
ICU direct after surgery	100 (25.8%)	12,408 (56.8%)
Proportion admitted directly to ICU after laparotomy		
Postop risk > 5%	75 (25.7%)	10,442 (82.3%)
Postop risk > 10%	62 (32.6%)	5600 (87.6%)
Post-operative LOS		
Mean (SD)	12.7 (17.2)	15.1 (16)
Median (IQR)	8.1 (8.6)	10 (12)
Unplanned return to theatre		
Yes	55 (8.7%)	1779 (8.1%)
No	576 (91.0%)	19,859 (90.9%)
Failure to rescue	16 (29.1%)	280 (15.7%)
Post-operative mortality (30 day)	103 (16.3%)	1905 (8.7%)

ASA, American Society of Anaesthetists, ICU, intensive care unit; LOS, length of stay; SD, standard deviation; IQR, interquartile range

**Table 4:** Post-operative mortality, preoperative mortality risk and case ascertainment rate in each centre

No.	Total no.	Mortality	NELA % mortality risk (mean (SD))	Case ascertainment rate
1	96	22.9%	13.5 (16.3)	>90%
2	70	20.0%	12.3 (17.0)	80–90%
3	68	7.4%	7.5 (11.7)	80–90%
4	70	15.7%	10.4 (16.1)	>90%
5	61	19.7%	5.2 (7.0)	>90%
6	64	18.8%	11.1 (17.1)	>90%
7	35	14.3%	5.5 (8.2)	80–90%
8	76	13.2%	15.6 (18.3)	>90%
9	28	21.4%	15.1 (20.5)	>90%
10	36	11.1%	5.9 (8.6)	80–90%
11	27	7.4%	7.4 (13.7)	>90%

NELA; national emergency laparotomy audit, SD; standard deviation

## Discussion

This study reports prospective data for patients undergoing emergency laparotomy in 11 hospitals of Greece over the period of 1.5 years. The main finding is the markedly high 30-day post-operative mortality of 16.3%. Factors associated with high risk of death were increasing age, ASA class, functional dependence, sepsis, diabetes mellitus and ascites. The rate of serious complications (class III and IV) was approximately 10%, and the most common complications were sepsis, septic shock, cardiac problems, and surgical site infections. Interestingly, a significant number of patients died in the ward without prior treatment in the ICU and only 12.6% of them were transferred from the ward to the ICU before they succumbed to post-operative complications. In comparison with contemporary data from the NELA study in the UK, there were no differences in the demographics, but more Greek patients presented with sepsis, cancer and required an immediate operation within 2 h. Notably, the timeliness of arrival in theatre was higher in the Greek study particularly among those requiring an immediate operation and this might be relevant with the observation that more British patients had a preoperative scan reaching 92.5%. The distribution of surgical operations was slightly different between the two cohorts, but the preoperative estimated mortality risk was similar. A striking difference was observed in the rates of patients who were treated in the ICU immediately after the operation. More than half British patients were transferred to the ICU post-operatively compared with less than a quarter of their Greek counterparts. The post-operative mortality of this study was almost double of that reported in NELA.

There are some limitations to this study. The sample is not population based but it includes mostly university and tertiary hospitals, so the results might not be applicable to the general population. Not every possible risk factor was captured but only well recognised ones from the literature. We did not include intraoperative haemodynamic parameters, volumes of fluids, transfusions of blood products and vasoconstrictors which have been associated with post-operative morbidity in other reports [18]. Therefore, unmeasured characteristics of the patients, the diseases or system processes may potentially explain the variation observed in mortality. For preoperative risk assessment, the NELA risk calculator was used which has been developed and validated in the UK population, but it has not been tested extensively in other populations such as ours. Therefore, risk assessment in this study might not be accurate.

The higher post-operative mortality rate in this study compared with NELA could be due to differences in the distribution of well-known measured prognostic variables such as sepsis, urgency and type of surgery, diagnosis of cancer, other unknown prognostic factors or simply due to random variation. The presence of a consultant surgeon and anaesthetist in the operating theatre is mandatory by law in Greece and should not contribute to the difference in the operative outcomes. However, we observed that significantly more patients died post-operatively than those who survived a serious post-operative complication grade III/IV (16.3% vs. 10.9%). Could this be due to offering surgical treatment to patients who have smaller or almost no chance to survive compared with NELA? The comparison of preoperative risk using the NELA calculator does not confirm this notion because it revealed similar risk rates between the two populations. It could be due to failure to recognise and treat timely post-operative complications. Indeed, post-operative mortality in this study was higher than the rate of survival from serious post-operative complications. More than 40% of the patients who died did not receive any treatment in the ICU and only 12.6% of them were transferred to the ICU after initial post-operative treatment in the ward before their death. This is also supported by the finding that the rates of patients who were transferred to the ICU directly after the operation was markedly lower compared with patients of NELA report in the UK. Even though reports on post-operative care of patients after emergency laparotomy in other countries are sparse, there is evidence from a multicentre study in Denmark that failure to allocate patients to the appropriate level of care immediately after surgery may contribute independently to the high post-operative mortality [19]. In this study, like ours, 84% of the patients were admitted post-operatively in the standard ward and mortality reached 18.5%. Similar results have been reported by Clark et al. in a smaller single centre study from the UK [11].

Has this trend towards less allocation of patients to ICU directly after emergency laparotomy been driven by contemporary pressures in the hospitals due to COVID-19? Unfortunately, we did not collect data on the local availability of ICU beds at the time of surgery. However, when we analysed post-operative pathways before and after the full eruption of the pandemic in Greece, we noticed that less patients (by 6.1%, *p* = 0.03) were admitted directly in the ICU during the pandemic than beforehand but this was not associated with any differences in post-operative mortality (Supplementary Table S9). Finally, it is difficult to explain the observed differences in the allocation of ICU beds to discrepancies in the general availability of critical care beds between countries. In European reports published well before and during the pandemic, there were similar numbers of critical care beds per 100 000 population between Denmark, the UK and Greece (approx. 6 per 100 000) [20, 21]. Thus, perhaps it is more about a culture for rationale allocation of hospital resources to those who are at highest risk rather than their general availability in the health care system [22].

Regarding the external validity of the results of this study, the possibility of selection bias as stated previously should always be considered. However, we used common inclusion criteria and definitions of variables with NELA which is the largest population-based database on emergency laparotomy in Europe. We used similar study design which is prospective registration of consecutive patients by treating clinicians to increase the accuracy and the completeness of data entry and we accomplished high rates of case ascertainment. The similarity of our sample with the population-based sample of NELA in terms of patients’ demographics and preoperative risk factors suggests that our results might be generalisable to the general population.

Improved preoperative risk stratification is a critical prerequisite for effective critical care involvement in the management of EL patients [7]. Risk calculators which have been developed in UK and USA populations such as NELA, the ACS-Risk calculator and POTTER should be evaluated and validated in other populations such as the Greek population [23–25]. The ongoing prospective multicentre RISK study (NCT04615520) aims to assess the most common surgical risk prediction tools in Greece. The Greek hospitals in their vast majority have IT systems which can easily incorporate electronic operating theatre booking applications with risk prediction tools. These systems serve not only as objective risk documentation tools but also as a trigger for mandatory multidisciplinary discussion of the perioperative management.

Finally, the results of this study point out ample room for improvement in the field of emergency laparotomy in this country. There is strong evidence from prospective trials that the establishment of consultant-led emergency surgical services, the introduction of multidisciplinary perioperative protocols and evidence-based quality improvement care bundles in conjunction with continuous education and raising awareness of healthcare staff has been associated with improved provision of care, timely management, improved clinical outcomes and reduced hospital costs [8, 9, 26, 27]. Towards these directions, NELA has established key 10 key performance indicators which are as follows: timely preoperative report of a CT scan by consultant radiologist, preoperative mortality risk calculation, timely arrival of patients in theatre according to the degree of urgency, presence or consultant surgeon and anaesthetist when the calculated mortality risk exceeds 5%, direct admission in the ICU when the calculated mortality exceeds 10% and perioperative geriatric assessment of patients aged 65 and older. Even though not all these indicators are based on high level evidence, they represent reasonable standards in quality improvement projects aiming to improve the care of emergency laparotomies [12].

## Supplementary Information

Below is the link to the electronic supplementary material.

Supplementary file1 (DOCX 228 kb)
