# Supplementary material for: The Hellenic Emergency Laparotomy Study (HELAS): A Prospective Multicentre Study on the Outcomes of Emergency Laparotomy in Greece
Source: World J Surg. 2022 Sep 15;47(1):130–9. doi: 10.1007/s00268-022-06723-6 (PMC9483423; doi:10.1007/s00268-022-06723-6)
Supplement: Supplementary file 1 — Supplementary file1 (DOCX 228 kb) [file 268_2022_6723_MOESM1_ESM.docx]

Supplement

Table of Contents

[Supplementary Table S1. The Strengthening the Reporting of Observational Studies in Epidemiology (STROBE) Statement. Complete checklist that should be included in cohort studies. 2](#_Toc112196548)

[Inclusion criteria: 5](#_Toc112196549)

[Exclusion criteria: 5](#_Toc112196550)

[Supplementary Table S2. Definitions of variables. 5](#_Toc112196551)

[Supplementary Figure S1. 9](#_Toc112196552)

[Supplementary Table S3. 30day postoperative mortality according to preoperative diagnosis and surgical procedure. 10](#_Toc112196553)

[Supplementary Table S4. Mortality associated with postoperative complications. 11](#_Toc112196554)

[Supplementary Table S5. Postoperative mortality by postoperative pathway of 633 patients undergoing emergency laparotomy. 11](#_Toc112196555)

[Supplementary Table S6. Distribution of NELA risk among non-survivors who were not treated in the ICU postoperatively. 1](#_Toc112196557)1

[Supplementary Table S7. Distribution of NELA calculated risk mortality between different postoperative pathways of 633 patients undergoing emergency laparotomy. 1](#_Toc112196556)2

[Supplementary Table S8. Independent risk factors of 30-day postoperative mortality ^(1)^ 12](#_Toc112196558)

[Supplementary Table S9. Postoperative ICU treatment and NELA mortality risk by admission date prior versus during full eruption of COVID-19 pandemic in Greece. 13](#_Toc112196559)

# Supplementary Figure S1.

Smoothed locally weighted regression lines (lowess) of the associations of age and body mass index with 30-day postoperative mortality on the log-odds scale (left) and on the probability scale (right). Entering an untransformed continuous variable into a logistic regression model assumes linearity on the log-odds scale. The graphs show that a linear relationship with the log odds of 30-day mortality is a good approximation for age, but for body mass index a U-shaped relationship is evident. Therefore, age was modelled on its original continuous scale, but body mass index was modelled using restricted cubic splines with four knots chosen at the 5th, 35th, 65th and 95th percentiles.

# Supplementary Table S1. The Strengthening the Reporting of Observational Studies in Epidemiology (STROBE) Statement. Complete checklist that should be included in cohort studies.

|  | Item No | Recommendation | Page No |  |
| --- | --- | --- | --- | --- |
| Title and abstract | 1 | (a) Indicate the study’s design with a commonly used term in the title or the abstract | 1 |  |
|  |  | (b) Provide in the abstract an informative and balanced summary of what was done and what was found | 3 |  |
| Introduction | | | | |
| Background/rationale | 2 | Explain the scientific background and rationale for the investigation being reported | 4 |  |
| Objectives | 3 | State specific objectives, including any prespecified hypotheses | 4 |  |
| Methods | | | | |
| Study design | 4 | Present key elements of study design early in the paper | 4,5 |  |
| Setting | 5 | Describe the setting, locations, and relevant dates, including periods of recruitment, exposure, follow-up, and data collection | 4,5 |  |
| Participants | 6 | (a) Give the eligibility criteria, and the sources and methods of selection of participants. Describe methods of follow-up | 4 & S2 |  |
|  |  | (b) For matched studies, give matching criteria and number of exposed and unexposed |  |  |
| Variables | 7 | Clearly define all outcomes, exposures, predictors, potential confounders, and effect modifiers. Give diagnostic criteria, if applicable | 4 & S3 |  |
| Data sources/ measurement | 8* | For each variable of interest, give sources of data and details of methods of assessment (measurement). Describe comparability of assessment methods if there is more than one group | 4 & S4 |  |
| Bias | 9 | Describe any efforts to address potential sources of bias | 5 |  |
| Study size | 10 | Explain how the study size was arrived at | 5 |  |
| Quantitative variables | 11 | Explain how quantitative variables were handled in the analyses. If applicable, describe which groupings were chosen and why | 5 |  |
| Statistical methods | 12 | (a) Describe all statistical methods, including those used to control for confounding | 5,6 |  |
|  |  | (b) Describe any methods used to examine subgroups and interactions | 5,6 |  |
|  |  | (c) Explain how missing data were addressed | 5,6 |  |
|  |  | (d) If applicable, explain how loss to follow-up was addressed | NA |  |
|  |  | (e) Describe any sensitivity analyses | NA |  |
| Results | | |  | |
| Participants | 13* | (a) Report numbers of individuals at each stage of study—eg numbers potentially eligible, examined for eligibility, confirmed eligible, included in the study, completing follow-up, and analysed | NA |  |
|  |  | (b) Give reasons for non-participation at each stage | NA |  |
|  |  | (c) Consider use of a flow diagram | NA |  |
| Descriptive data | 14* | (a) Give characteristics of study participants (eg demographic, clinical, social) and information on exposures and potential confounders | 6 & Table 1 |  |
|  |  | (b) Indicate number of participants with missing data for each variable of interest | 6 & Table 1 |  |
|  |  | (c) Summarise follow-up time (eg, average and total amount) | NA |  |
| Outcome data | 15* | Report numbers of outcome events or summary measures over time | Table 1,2 |  |

| Main results | 16 | (a) Give unadjusted estimates and, if applicable, confounder-adjusted estimates and their precision (eg, 95% confidence interval). Make clear which confounders were adjusted for and why they were included | Table 1, Table S2 |
| --- | --- | --- | --- |
|  |  | (b) Report category boundaries when continuous variables were categorized | Table 1 Figure S1 |
|  |  | (c) If relevant, consider translating estimates of relative risk into absolute risk for a meaningful time period | NA |
| Other analyses | 17 | Report other analyses done—eg analyses of subgroups and interactions, and sensitivity analyses | Supplement |
| Discussion | | | |
| Key results | 18 | Summarise key results with reference to study objectives | 8 |
| Limitations | 19 | Discuss limitations of the study, taking into account sources of potential bias or imprecision. Discuss both direction and magnitude of any potential bias | 9 |
| Interpretation | 20 | Give a cautious overall interpretation of results considering objectives, limitations, multiplicity of analyses, results from similar studies, and other relevant evidence | 9 |
| Generalisability | 21 | Discuss the generalisability (external validity) of the study results | 10 |
| Other information | | | |
| Funding | 22 | Give the source of funding and the role of the funders for the present study and, if applicable, for the original study on which the present article is based | 11 |

*Give information separately for exposed and unexposed groups.

NA, not applicable.

# Inclusion criteria:

- Age >18yrs
- Emergency laparotomy (operation simultaneously with resuscitation usually within one hour) or urgent (operation as soon as possible after resuscitation, within 24hrs)
- Operation in the gastrointestinal tract:
- Open or laparoscopic, or laparoscopically assisted procedures.
- Procedures involving the stomach, small or large bowel, or rectum for conditions such as perforation, ischaemia, abdominal abscess, bleeding or obstruction
- Wash out/evacuation of intraperitoneal abscess or haematoma
- Bowel resection/repair due to incarcerated/incisional hernias
- Bowel resection or repair due to incarcerated umbilical, inguinal or femoral hernias
- Open or laparoscopic adhesiolysis
- Laparotomy/laparoscopy with inoperable pathology
- Return to theatre for repair of a substantial dehiscence of major abdominal wound (i.e. “burst abdomen”)
- Return to theatre after any operation (including vascular, gynaecology, urology, cardiac) meeting the criteria above
- In the case of multiple procedures in the abdominopelvic cavity the patient is included if the main procedure is a general surgical one (i.e. if bowel resection happens during an open aneurysm repair it should not be included)
- Any intra-abdominal procedure not identifiable within exclusion criteria should be included.

# Exclusion criteria:

- Patients under 18
- Elective operation
- Diagnostic laparoscopy or laparotomy where no other procedure is performed (NB, if no procedure is performed due to inoperable pathology, then include)
- Appendicectomy with or without drainage of localized abscess
- Cholecystectomy with or without drainage of localized abscess
- Hernia repair without bowel resection
- Minor abdominal wound revision
- Vascular surgery
- Gynaecological surgery – c-section – ruptured ectopic pregnancy
- Surgery relating to organ transplantation

# Supplementary Table S2. Definitions of variables.

| Variable | Definition |
| --- | --- |
| Smoker | The patient has smoked cigarettes in the year prior to admission for surgery. |
| Chronic Steroids use | Regular administration of oral or parenteral corticosteroid medications or immunosuppressants for a chronic medical condition, within the 30 days prior to surgery, or at the time the patient is being considered as a candidate for surgery. |
| Sepsis within 48hrs preop. | Any of the following occurring within 48 hours prior to surgery:  SIRS: Systemic Inflammatory Response Syndrome (SIRS)  Sepsis: life-threatening organ dysfunction due to a dysregulated host Septic shock response to infection, organ dysfunction is defined as an increase of 2 points or more in the Sequential Organ Failure Assessment (SOFA) score.  Sepsis and (despite adequate volume resuscitation) both of: Persistent hypotension requiring vasopressors to maintain MAP greater than or equal to 65 mm Hg, and Lactate greater than or equal to 2 mmol/L. |
| Patient intubated preop. | A patient requiring ventilator-assisted respiration at any time during the 48 hours preceding surgery. |
| Presence of malignancy | Estimate of extent of intra-abdominal malignancy based on available radiological imaging and clinical experience. This variable has the following categories: No malignancy, primary tumour, lymph node metastasis, distant metastasis |
| Diabetes mellitus | The individual requires daily dosages of exogenous parenteral insulin or an oral hypoglycemic agent to prevent a hyperglycemia. |
| Ascites 30days prior surgery | The presence of fluid accumulation in the peritoneal cavity noted on physical examination, abdominal ultrasound, or abdominal CT/MRI within 30 days prior to surgery. Documentation must state either active or a history of liver disease or must state secondary to malignancy. |
| ASA class | American Society of Anesthesiologists class:  I: Normal healthy patient.  II: Patient with mild systemic disease.  III: Patient with severe systemic disease.  IV: Patient with severe systemic disease that is a constant threat to life.  V: Moribund patient who is not expected to survive without the operation. |
| EGG findings | Findings on preoperative electrocardiogram. This variable has the following categories. Normal, AF 60-90bpm, AF>90bpm, other arrhythmia, Q, ST, T abnormality |
| Cardiac comorbidity | Diuretic, digoxin, antianginal or antihypertensive therapy, Peripheral oedema, warfarin therapy, Raised jugular venous pressure or cardiomegaly on CXR |
| Dyspnea history | The patient's dyspnea status when they were in their usual state of health, prior to the onset of the acute illness. This variable has the following categories: dyspnea on exertion, dyspnea at rest or long-term oxygen therapy (LTOT) |
| Hemodialysis or CVVF | Acute or chronic renal failure requiring treatment with peritoneal dialysis, hemodialysis, hemofiltration, hemodiafiltration, or ultrafiltration within 2 weeks prior to surgery. |
| Preop acute renal failure | A clinical condition associate with rapid decline of kidney function. The patient meets one of the following:  -Increased BUN on two measurements AND two Cr results > 3mg/dl  -Surgeon or physician has documented Acute Renal Failure AND one of the following: Increased BUN on two measurements OR Two Cr results > 3mg/dl. |
| Functional status | The best functional status/level of self-care demonstrated by the patient within the 30 days prior surgery  Independent: The patient does not require assistance from another person for any activities of daily living. This includes a person who can function independently with prosthetics, equipment, or devices.  Partially dependent: The patient requires some assistance from another person for activities of daily living.  Totally dependent: The patient requires total assistance for all activities of daily living. |
| Urgency of operation | Based on surgeon’s clinical experience, this is the maximum time the patient could reasonably wait for surgery. This is categorized as:  Expedited (>18 hours), Urgent (6-18 hours), Urgent (2-6 hours), Immediate (<2 hours) |
| Operative severity | Operative severity of intended surgical intervention  Major plus:  -All colonic resections (excluding colostomies alone)  -all gastrectomies (but not repair perforated or bleeding ulcer)  -Small bowel tumour resection  -Re-operations for ongoing sepsis or bleeding  -Laparostomy  -Intestinal bypass  Major:  All other procedures including:  -Stoma formation  -Small bowel resection  -Adhesiolysis  -Perforated or bleeding ulcer |
| CT preoperatively | Was a CT done up to 48hrs prior the operation. |
| Postoperative pathway | Ward care: Patient admitted in ward postoperatively until discharge  Ward care prior ICU: Patient admitted in ward postoperatively, then to the ICU  ICU direct after surgery: Patient admitted directly to the ICU postoperatively |
| Return to OR | Patient undergoing an operation for the management of a complication of the emergency laparotomy. |
| Complication severity | I: Any deviation from the normal postoperative course without the need for pharmacological treatment or surgical, endoscopic, and radiological interventions Allowed therapeutic regimens are drugs as antiemetics, antipyretics, analgesics, diuretics and electrolytes and physiotherapy. This grade also includes wound infections opened at the bedside.  II: Requiring pharmacological treatment with drugs other than such allowed for grade I complications. Blood transfusions and total parenteral nutrition are also included.  III: Requiring surgical, endoscopic, or radiological intervention  IV: Life-threatening complication (including CNS complications) requiring IC/ICU-management  V: Death of a patient |
| Sepsis | - Life-threatening organ dysfunction due to a dysregulated host response to infection, organ dysfunction is defined as an increase of 2 points or more in the Sequential Organ Failure Assessment (SOFA) score. |
| Septic shock | - Sepsis and (despite adequate volume resuscitation) both of: Persistent hypotension requiring vasopressors to maintain MAP greater than or equal to 65 mm Hg, and Lactate greater than or equal to 2 mmol/L. |
| Bleeding req transfusion | Patient requires transfusion of blood products (RBCs, FFP or PLTs) |
| Cardiac arrest | Documented cardiac arrest requiring CPR |
| Pneumonia | Lower respiratory tract infection based on clinical, radiological and laboratory data |
| Pulmonary embolism | Diagnosis based either on CTPA or ventilation/perfusion (V/Q) scan. |
| Stroke | Ischemic or hemorrhagic stroke, diagnosis by neurologist based on clinical and radiological data |
| Acute kidney injury | A clinical condition associate with rapid decline of kidney function. The patient meets one of the following:  -Increased BUN on two measurements AND two Cr results > 3mg/dl  -Surgeon or physician has documented Acute Renal Failure AND one of the following: Increased BUN on two measurements OR 2 Cr results > 3mg/dl. |
| Myocardial Infarction | Diagnosis by cardiologist based on the combination of clinical signs and symptoms, ECG and cardiac enzymes |
| Surgical Site Infection grade | Superficial: Skin or subcutaneous tissue is involved, occurs within 30 days postoperatively, and must fulfill one of the following additional criteria: purulent drainage from incision with or without diagnostic laboratory testing (culture), isolated organisms from aseptically obtained fluid or tissue culture in incision at least one sign or symptom of clinical infection: localized pain, edema, erythema, warmth and the superficial incision is deliberately opened by a surgeon (unless culture of incision is negative) diagnosis of a superficial incisional SSI by a surgeon or attending physician  Deep: Involves deep soft tissues such as fascia or muscle within incision, occurs within 30 days postoperatively without implant, occurs within 1 year if implant is in place and infection appears to be directly related to surgical procedure, and must fulfill one of the following additional criteria: purulent drainage from incision but not from the organ/space of the site dehiscence or deliberate opening by the surgeon from the deep incision when the patient has at least one of the following signs or symptoms of clinical infection (fever greater than 100.4°F, localized pain or edema, unless culture is negative), abscess or other evidence of infection involving the deep incision is found during examination of incision, reoperation, or pathologic or radiologic exam, diagnosis of a deep incisional SSI by a surgeon or attending physician  Organ/space: Involves any part of the anatomy other than the incision, occurs within 30 days postoperatively without implant, occurs within 1 year if implant is in place and infection appears to be directly related to surgical procedure, and must fulfill one of the following: purulence from a drain that was placed via stab incision into the organ/space (infection of drain site is not an SSI), isolated organisms from aseptically obtained fluid or tissue from the organ/space, abscess or other evidence of infection involving the deep incision is found during examination of incision, reoperation, or pathologic or radiologic exam, diagnosis of an organ/space SSI by a surgeon or attending physician |
| Deep Venous Thrombosis | Diagnosis set by ultrasonography of the affected limb |
| Urinary tract infection | Diagnosis based on clinical signs, laboratory data and positive urine culture |
| Delirium | Acute and fluctuating disturbance of consciousness with reduced ability to focus, maintain, or shift attention, accompanied by change in cognition and perceptual disturbances secondary to a general medical condition, diagnosis set by psychiatrist |
| Other | Other complication other than the aforementioned |

# Supplementary Table S3. 30day postoperative mortality according to preoperative diagnosis and surgical procedure.

|  | N died | N cohort | Mortality % |
| --- | --- | --- | --- |
| Diagnosis |  |  |  |
| Perforation | 38 | 225 | 16.9 |
| Obstruction | 29 | 247 | 11.7 |
| Ischemia | 20 | 94 | 21.3 |
| Other | 16 | 65 | 24.6 |
| Procedure |  |  |  |
| Adhesiolysis | 3 | 75 | 4.0 |
| Small bowel resection | 24 | 130 | 18.5 |
| Colectomy right | 10 | 58 | 17.2 |
| Hartmann's procedure | 14 | 73 | 19.2 |
| Strangulated hernia with bowel resection | 3 | 38 | 7.9 |
| Peptic ulcer repair | 12 | 75 | 16.0 |
| Colectomy other | 12 | 50 | 24.0 |
| Stoma formation | 8 | 41 | 19.5 |
| Other | 17 | 91 | 18.7 |

Missing values <1.5%

# Supplementary Table S4. Mortality associated with postoperative complications.

| **Postoperative outcome** | **No. of patients** | **Proportion (%)** | **Mortality within complications** |
| --- | --- | --- | --- |
| Return to theatre | 55 | 8.7% | 16 (29.1%) |
| Sepsis | 90 | 14.3% | 41 (45.6%) |
| Septic shock | 63 | 10.0% | 44 (69.8%) |
| Bleeding requiring transfusion | 38 | 6.0% | 16 (42.1%) |
| Cardiac arrest | 56 | 8.9% | 51 (91.1%) |
| Pneumonia | 45 | 7.1% | 12 (26.7%) |
| Pulmonary embolism | 4 | 0.6% | 0 |
| Stroke | 1 | 0.2% | 0 |
| Acute kidney injury | 45 | 7.1% | 25 (55.6%) |
| Myocardial infarction | 4 | 0.6% | 1 (25.0%) |
| Surgical site infection |  |  |  |
| Superficial incisional | 46 | 59.0% | 1 (2.2%) |
| Deep incisional | 20 | 25.6% | 3 (15.0%) |
| Organ/space | 12 | 15.4% | 4 (33.3%) |
| Deep venous thrombosis | 1 | 0.2% | 1 (100%) |
| Urinary tract infection | 5 | 0.8% | 0 |
| Delirium | 19 | 3.0% | 7 (36.8%) |
| Other complication | 83 | 13.2% | 28 (33.7%) |

# Supplementary Table S5. Postoperative mortality by postoperative pathway of 633 patients undergoing emergency laparotomy.

| Postoperative pathway | Postoperative death within 30 days | |
| --- | --- | --- |
|  | Yes  N (%) | No  N (%) |
| Ward care | 45 (43.7%) | 465 (88.1%) |
| Surgery 🡪 ICU | 40 (38.8%) | 51 (9.7%) |
| Ward 🡪 ICU | 13 (12.6%) | 9 (1.7%) |
| ICU 🡪 surg 🡪 ICU | 5 (4.9%) | 3 (0.6%) |

Missing values 2(0.3%)

# Supplementary Table S7. Distribution of NELA calculated risk mortality between different postoperative pathways of 633 patients undergoing emergency laparotomy.

| Postoperative pathway | NELA calculated mortality risk | | |
| --- | --- | --- | --- |
|  | Low (<5%)  N(%) | High (5-9.9%)  N(%) | Very high (>9.9%)  N (%) |
| Ward care | 307 (91.6%) | 85 (82.5%) | 115 (60.5%) |
| Surgery🡪 ICU | 22 (6.6%) | 12 (11.7%) | 57 (30.5%) |
| Ward🡪ICU | 4 (1.2%) | 5 (4.9%) | 13 (6.8%) |
| ICU🡪surgery🡪 ICU | 2 (0.6%) | 1 (1.0%) | 4 (2.1%) |

Missing values 11 (1.7%)

NELA: National Emergency Laparotomy Audit

# Supplementary Table S6. Distribution of NELA risk among non-survivors who were not treated in the ICU postoperatively.

|  | N(%) |
| --- | --- |
| Age (years) |  |
| <55 | 2 (4.4) |
| 55-65 | 3 (6.7) |
| 65-75 | 10 (21.2) |
| 75-85 | 16 (35.6) |
| >85 | 14 (31.1) |
| Total | 45 (100.0) |
| NELA risk |  |
| <5% | 6 (13.0) |
| 5-10% | 6 (11.1) |
| >10% | 33 (73.3) |
| Missing | 1 (2.2) |
| Total | 45 (100.0) |
| Survival (days) |  |
| Median(IQR) | 4.7(9.7) |
| Range | 0.06-34 |

NELA, National Emergency Laparotomy Risk; IQR, interquartile

# Supplementary Table S8. Independent risk factors of 30-day postoperative mortality ^(1)^

| Risk factor | aOR | 95% CI | P value |
| --- | --- | --- | --- |
| Age, years (effect per 10 years increase) | 1.60 | 1.28 - 2.01 | 0.000 |
| ASA class |  |  |  |
| I-II | 1.00 (ref.) |  |  |
| III | 1.79 | 0.89 - 3.62 | 0.082 |
| IV-V | 6.92 | 3.25 – 14.74 | 0.000 |
| Dependent preoperative functional status | 2.04 | 1.15 - 3.61 | 0.017 |
| Ascites | 2.16 | 1.11 - 4.21 | 0.022 |
| Sepsis within 48h from surgery |  |  |  |
| None (ref.) | 1.00 (ref.) |  |  |
| Two SIRS criteria | 1.28 | 0.65 - 2.51 | 0.453 |
| Severe sepsis or Septic Shock | 4.83 | 2.29 – 10.18 | 0.000 |
| Diabetes mellitus | 1.87 | 1.02 - 3.41 | 0.041 |

aOR, adjusted odds ratio; CI confidence interval; ASA, American Society of Anesthesiologists; SIRS, systemic inflammatory response syndrome.

Note.

^(1)^ The table reports the results of fitting a multivariable mixed-effects logistic regression with hospital entered as random intercept. The final set of risk factors was selected following purposeful variable selection from 19 candidate variables. Predetermined criteria to retain a variable into the final model were p-value <0.05 and/or substantial confounding effect.

# Supplementary Table S9. Postoperative ICU treatment and NELA mortality risk by admission date prior versus during full eruption of COVID-19 pandemic in Greece.

|  | Admission date* | |  |
| --- | --- | --- | --- |
|  | “pre-pandemic”  1/1/2020-1/11/2020 | “pandemic”  1/11/2020-1/5/2021 | P** |
| Postop pathway | N(%) | N(%) | 0.033 |
| ward care | 259(77.8) | 251(84.2) |  |
| ward 🡪 ICU | 14(4.2) | 8(2.7) |  |
| surgery 🡪 ICU | 58(17.4) | 33(11.1) |  |
| ICU 🡪 surg 🡪 ICU | 2(0.6) | 6(2.0) |  |
| Total | 333 | 298 |  |
| NELA mortality risk |  |  |  |
| <5.00% | 185(55.6) | 147(49.3) | 0.295 |
| 5.00-9.99% | 51(15.3) | 51(17.1) |  |
| ≥10.00% | 93(27.9) | 96(32.2) |  |
| Missing | 4(1.2) | 4(1.3) |  |
| Postop death 30day |  |  |  |
| Yes | 53(15.9) | 49(16.4) | 0.941 |
| No | 279(83.8) | 248(83.2) |  |
| Missing | 1(0.3) | 1(0.3) |  |

*According to data from the Greek government, in November 2020 the availability of ICU beds was reduced for the first time to less than 20% in some areas of Greece (data from: <https://covid19.gov.gr/24i-ekthesi-proodou-paratiritiriou/> accessed on 8.8.2022)

**Chi square

NELA: National Emergency Laparotomy Audit, ICU: Intensive Care Unit.
